# No colonization resistance to *Campylobacter jejuni* in broilers fed brown algal extract-supplemented diets

**DOI:** 10.3389/fmicb.2024.1396949

**Published:** 2024-06-27

**Authors:** Eliška Eliasson, Li Sun, Gunnar Cervin, Henrik Pavia, Gustav Tällberg, Patrik Ellström, Emma Ivarsson

**Affiliations:** ^1^Department of Applied Animal Science and Welfare, Swedish University of Agricultural Sciences, Uppsala, Sweden; ^2^Department of Marine Sciences, Tjärnö, University of Gothenburg, Strömstad, Sweden; ^3^Zoonosis Science Center, Uppsala University, Uppsala, Sweden; ^4^Zoonosis Science Center, Department of Medical Sciences, Uppsala University, Uppsala, Sweden

**Keywords:** brown algae, *Saccharina latissima*, laminarin, *Campylobacter jejuni*, host specificity, microbiota, broiler

## Abstract

**Introduction:**

*Campylobacter jejuni* gastroenteritis is the most commonly reported zoonosis within the EU, with poultry products regarded as the primary source of transmission to humans. Therefore, finding strategies to reduce *Campylobacter* colonization in broilers holds importance for public health. Recent studies suggest that supplementation of broiler feed with brown algal extracts, particularly laminarin, can provide beneficial effects on broiler gut health, growth performance, and gut microbiota. However, its effect on gut microbiota development and subsequent reduction of *Campylobacter* loads in broiler caeca during the later stages of the birds' lives remains unclear.

**Methods:**

Experimental colonization of Ross 308 broilers with two different strains of *C. jejuni* was conducted, with groups fed either a basal diet or the same basal diet supplemented with 725 ppm algal extract from *Saccharina latissima* to provide 290 ppm laminarin. Fecal samples were collected for bacterial enumeration, and caecal samples were obtained before and after the *C. jejuni* challenge for the determination of microbiota development.

**Results and discussion:**

No significant differences in fecal *C. jejuni* concentrations between the groups fed different diets or exposed to different *C. jejuni* strains were observed. This suggests that both strains colonized the birds equally well and that the laminarin rich algal extract did not have any inhibitory effect on *C. jejuni* colonization. Notably, 16S rRNA amplicon sequencing revealed detailed data on the caecal microbiota development, likely influenced by both bird age and *C. jejuni* colonization, which can be valuable for further development of broiler feed formulations aimed at promoting gut health.

## 1 Introduction

*Campylobacter jejuni* (*C. jejuni*) is a common gram-negative bacterial pathogen associated with foodborne illnesses in humans, and broiler chickens are known to be a significant reservoir for this bacterium (EFSA, 2020). Finding strategies to reduce *Campylobacter* colonization in broiler flocks is important for food safety and public health.

*Saccharina latissima* is a brown algae belonging to the family Laminariaceae and is rich in complex polysaccharides such as alginate, laminarin, and fucoidan (Sharma et al., 2018). It has been proposed that laminarin could have prebiotic potential (Cherry et al., 2019). A study conducted on broiler chicks, where birds were fed a laminarin-rich extract from *Laminaria spp*. at 300 ppm, found that it exhibited promising effects on growth performance, protective immune responses, and beneficial effects on the gastrointestinal microbial profile (Venardou et al., 2021). Additionally, an increase in the expression of the cytokine interleukin 17A (IL17A) in the broiler's duodenum was observed. Interleukin 17A has been reported to be involved in the immune response against several infectious agents, including *Campylobacter* (Connerton et al., 2018). Sweeney and O'Doherty (2016) reported that supplementing the basal diet with laminarin at a concentration of 250 ppm during the initial stages of the chickens' life led to notable improvements in villus morphology and enhanced tight junction integrity in Ross 308 broilers. However, the same study reported no significant differences in *C. jejuni* colonization during experimental colonization (day three post-hatch to day 13) between broilers raised on an algal extract-supplemented diet and those raised on a conventional diet in the post-hatch period. Yet, the investigation of algal extracts' impact on the microbiota and *C. jejuni* bacterial load in broiler caeca during later stages of life remains unexplored in current scientific literature.

Although the epizootology of *C. jejuni* and its spread into broiler production is still not fully understood, it is well-known that wild birds constitute a natural reservoir for this pathogen (French et al., 2009; Waldenström et al., 2010; Griekspoor et al., 2013; Mourkas et al., 2022). Therefore, it is also likely that they play a role in the transmission of *C. jejuni* between wild animals, to domestic production, and further to humans. In wild birds, *C. jejuni* shows strong host specificity such that different bird taxa carry different genotypes of *C. jejuni* (Griekspoor et al., 2013). This specificity does not seem to correlate with the diet or habitat of the birds but rather with specific species associated microbiota. In a previous experiment, the relative colonization ability in mallard ducks (*Anas platyrhynchos*) was compared between *C. jejuni* strains of different host origins (Atterby et al., 2018). Not surprisingly, *C. jejuni* of mallard origin colonized the birds best. However, a strain from a song thrush (*Turdus philomelos*) was a very poor colonizer and could not establish long-term colonization in this species, whereas *C. jejuni* isolated from a broiler chicken could colonize the mallards. A similar experiment was previously performed on wild robins (*Erithacus rubecula*), where a song thrush strain of the same ST-type could colonize the birds for an average of 6.8 days whereas a strain isolated from a human could not establish colonization (Waldenström et al., 2010). These studies suggest that wild bird species might exhibit colonization resistance to *C. jejuni* genotypes that originate from distantly related hosts. On the other hand, there also exist generalist genotypes of *C. jejuni*, that can be found in many different host species (Gripp et al., 2011), and hence, colonization resistance does not seem to apply to such strains. Broiler chickens are known to harbor many different genotypes of *C. jejuni* and it is not known if this host species also shows colonization resistance to *C. jejuni* adapted to distantly related hosts.

The present study aimed to examine the impact of feed supplementation with a laminarin-rich algal extract from *Saccharina latissima* (*AE*) on the development of the caecal microbiota in broilers and assess its influence on resistance to intestinal colonization with *C. jejuni* upon experimental colonization. Groups of Ross 308 chickens were intraoesophagically inoculated at 17 days of age with two different *C. jejuni* strains, one of chicken origin and one isolated from a wild song thrush. Based on previous experiments, our hypothesis was that the *C. jejuni* strain isolated from chicken would exhibit superior colonization ability in broilers compared to the strain originating from song thrush and that any effect of the feed supplementation with *AE* would be more pronounced against the song thrush strain than the chicken strain.

## 2 Materials and methods

This study was conducted as part of a larger project where the effect of feeding brown algae (intact or as an *AE*) to broiler breeder hens was evaluated. Previously, the effect on hens' antibody response, egg quality, and the chick quality of their progeny was assessed (Ivarsson et al., 2023). In addition, the early development of hens' progenies with a main focus on early growth performance was examined (Supplementary Table 1). These progenies were subsequently utilized in the present study. The experiment was carried out at the Swedish Livestock Research Center of the Swedish University of Agricultural Sciences and was approved by the committee for animal ethics of the Uppsala region (approval number 5.8.18-10572/2019).

### 2.1 Housing

A total of 255 mixed-sex Ross 308 chicks were allocated into groups of 10-13 chickens in 24 experimental pens, the allocation of chicks to the rearing pens was determined prior to the hatching. The solid floor pens with dimensions 1.5 × 0.75 meters were bedded with wood shavings and equipped with feeders and nipple drinkers. The house temperature was initially set at 34°C during hatching (day 0) and then reduced to 33°C for the first 3 days. Thereafter, the temperature gradually decreased with age until reaching 23°C at day 24, remaining the same for the rest of the trial period. Lighting conditions consisted of a 24-h cycle during the first 2 days, after which the duration of light was reduced by 1 h daily until day eight, resulting in 18 h of light per day for the remaining period. All chickens were marked with color identification at hatch, which was replaced by a 1 cm × 1 cm laminated neck tag (Jolly Fine, Jolly, Italy) at 7 days of age. Two chickens from each replicate were used for the collection of fecal samples.

### 2.2 Experimental design and diets

The chicken facility with 24 experimental pens in total was divided into two parts, each consisting of 12 pens. Each part was split into two chick treatment groups: (1) basal diet formulated according to the nutrient requirement of Ross 308 (control - *C*), and (2) basal diet supplemented with 725 ppm algal extract (*AE*) sourced from *Saccharina latissima*. The supplemented diet was optimized to contain 290 ppm laminarin and was provided from the first day of the animal's life until the end of the trial. Both chick diets were pelleted and formulated according to the nutrient requirement of Ross 308 (Aviagen., 2019). The algae used in the study were cultivated at sea on longlines in the Koster archipelago, located outside Tjärnö Marine Biological Laboratory on the Swedish West Coast (Thomas et al., 2022). In the experiment, dried algae were utilized as a substrate for the *AE*, with the detailed processing procedures described in Ivarsson et al. (2023). The resulting *AE* contained 41.6 % laminarin, analyzed enzymatically by measuring the β 1,3/1,6-glucan content using the K-YBGL 12/16 assay kit from Megazyme. Additionally, the ash content of the *AE* was determined to be 14.5% on a dry matter (DM) basis. The non-starch polysaccharide content was analyzed by the Uppsala method (Theander et al., 1995), and amounted to a total of 45.7% on a DM basis. The specific composition of the polysaccharides was as follows: 31% glucose, 2.2% fucose, 3.0% mannose, 1.8% galactose, 0.7% xylose, and 0.5% arabinose.

### 2.3 *Campylobacter jejuni* inoculation

At 17 days of age, the bird group size was reduced to four chickens per pen, and chicks were intraoesophagically inoculated with 1 ml of a PBS solution containing ~10^2^ CFU of *C. jejuni* as determined by optical density at 405 nm. Two different *C. jejuni* strains were used and birds inoculated with each of the strains were kept apart in two different parts of the facility, separated by a flexible wall. *C. jejuni* strain #65 (ST-104, in ST-21 CC) was isolated from a broiler chicken in the UK and *C. jejuni* strain #3926 (ST-1315 in ST-1304 CCs) was isolated from a wild song thrush captured in Sweden. The bacterial concentration of the two inoculates was confirmed by CFU counts on blood agar plates after 24 h incubation at 42°C in a microaerobic atmosphere to be 2 x 10^2^ cfu/ml for *C. jejuni* strain #65 and 4 x 10^2^ cfu/ml for strain #3926.

### 2.4 Fecal and caecal samples collection

Fecal samples were taken from all birds 1 day before the inoculation, to ensure that the birds were culture-negative for *Campylobacter* before the challenge. Subsequently, samples were collected regularly throughout the experimental period at 1 day post-infection (dpi), 2 dpi, 3 dpi, 5 dpi, 7 dpi, 14 dpi, and 19 dpi for bacterial enumeration by culture on agar plates. Focal birds from each pen were placed individually in clean boxes and sterile cotton swabs were used to collect ~100 mg of fresh fecal matter per bird. Thereafter, samples were re-suspended in 1 ml Luria-Bertani (LB) medium complemented with 20% glycerol, followed by vortexing and centrifugation (100 × g for 15 s) for sedimentation of the fecal matter. Subsequently, six 20 μl aliquots were withdrawn from each sample and added to the first column of a 96-well plate containing 180 μl of PBS per well. The six replicates were serially diluted 10-fold and six dilution steps were plated in aliquots of 10 μl on modified charcoal cefoperazone deoxycholate agar (mCCDA) plates using an 8-channel pipette according to the 6 × 6 drop plate method described by Chen et al. (2003). Plates were left to dry for 10 min and incubated for 30 h at 42°C under microaerobic conditions (Campygen, Thermo Fisher, USA). After incubation, colonies were counted and a mean value was created from the six spots from the column that contained the highest number of well separated colonies. Results are presented for four treatments; chicken strain with control feed (Ch + *C*), chicken strain with algal extract (Ch + *AE*), song thrush strain with control feed (Th + *C*), and song thrush strain with algal extract (Th + *AE*).

Caecal samples were collected from two birds per pen at day 7, day 14, and day 37 of the experiment. Euthanasia was performed by administration of sodium pentobarbital intravenously through the wing vein. An aseptic procedure was followed to obtain caecal content, which was sampled into 2.0 ml screw cap microtubes (Sarstedt AG and Co, Germany) and immediately placed in liquid nitrogen. All samples were stored at−80°C until further analysis. The microbial composition of the caecal content was investigated by 16S rRNA amplicon sequencing.

### 2.5 DNA extraction, sequencing, and bioinformatics analysis

The DNA extraction of caecal digesta samples was conducted as previously described (Ivarsson et al., 2023). In brief, 400 microliters of ASL lysis buffer (Qiagen, Germany) were added to the thawed sample and homogenized. Thereafter, 120 μl suspension was used for bead beating on Precellys evolution homogenizer (Bertin Technologies SAS, France) at 8000 rpm for 2 × 60 sec with 30-s pauses. After centrifugation, 120 μl of the supernatant was used for DNA extraction using the EZ1 Advanced XL instrument (Qiagen, Germany) according to the manufacturer's instructions.

The caeca sample DNA extractions were sequenced at Novogene (Cambridge, UK), using the Illumina NovaSeq 6000 PE250 platform. The primer set targeting the V4 region of 16S rRNA gene 515F (GTGCCAGCMGCCGCGGTAA) and 806R (GGACTACHVGGGTWTCTAAT) were used for amplification. PCR reactions were performed with Phusion High-Fidelity PCR Master Mix (New England Biolabs, USA). PCR products (~400–450 bp) were separated by electrophoresis on 2% agarose gel, purified with a Qiagen Gel Extraction Kit (Qiagen, Germany), and pooled at equal concentrations. Sequencing libraries were generated using the NEBNext Ultra DNA Library Prep Kit (Illumina, USA), followed by quantification using Qubit (Thermo Fisher Scientific, USA) and sequencing.

The 16S rRNA gene sequencing data processing was performed as described in Sun et al. (2022), with the following modification: (1) using the truncation length of 221 bp for both forward and reverse reads; (2) the SILVA SSU Ref NR 99 138 dataset was used for taxonomic classification (Pedregosa et al., 2011); (3) the generalized UniFrac distance matrix (alpha = 0.5) was generated using the QIIME2 diversity plugin (Bolyen et al., 2019).

### 2.6 Statistical analysis

All statistical analysis was performed with R (R Core Team, 2019). To investigate the effect of dietary treatment and *Campylobacter* strain, Quasi-Poisson regression (Ver Hoef and Boveng, 2007) was employed to analyse the plate counting data for C. jejuni, while Tukey's Honestly Significant Difference (HSD) test was utilized to conduct multiple pairwise comparisons. For sequencing data, the number of observed ASVs was calculated from rarefied ASV table. The Kruskal-Wallis rank test, followed by Dunn's test for pairwise comparisons with Benjamini and Hochberg (B–H) correction, was used to check for statistically significant differences in observed ASVs between treatment groups, using the QIIME2 q2-diversity plugin (Kruskal, 1952; Dunn, 1964; Benjamini and Hochberg, 1995). Permutational multivariate analysis of variance (PERMANOVA) of generalized UniFrac distance matrix with B-H correction was conducted to evaluate differences between the dietary and *Campylobacter* treatment groups, using the q2-diversity plugin (Anderson, 2001). Mixed effects linear models were fitted and analyzed using R packages lme4 (Bates et al., 2015), lmerTest (Kuznetsova et al., 2017), pbkrtest (Halekoh and Højsgaard, 2014), and emmeans (Searle et al., 1980). Age, feed treatment, and *Campylobacter* strain were used as fixed effects, and pen as a random effect. *P* < 0.05 was considered significant.

## 3 Results

### 3.1 *Campylobacter* colonization not influenced by *AE*

**Figure 1:**
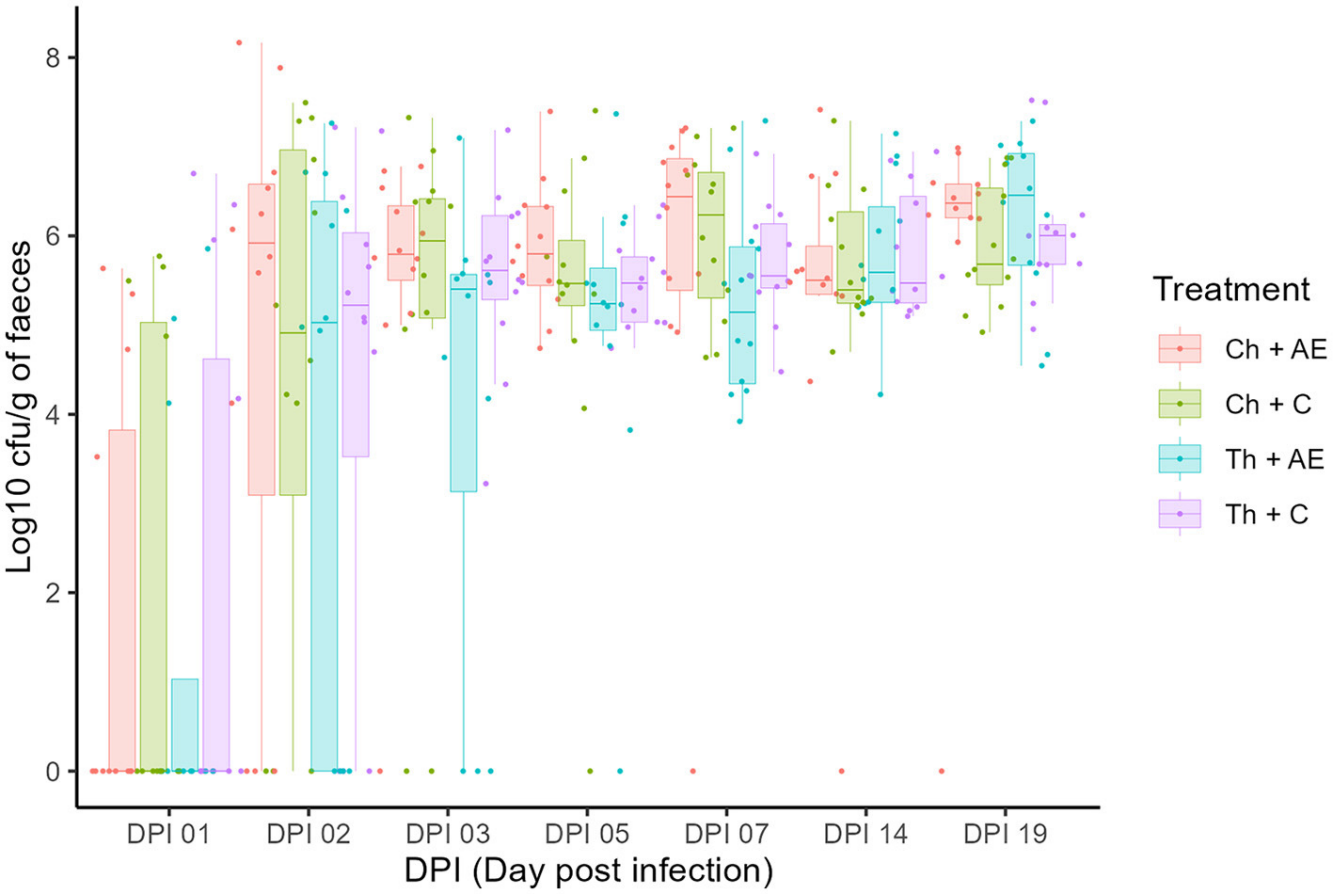
Colony forming unit counts of *Campylobacter jejuni* colonization in fecal samples at different dpi (days post-infection). Dots (data points) represent 10-log (CFU/ml) in individual fecal samples based on mean values of six replicate dots per plate at a given sampling point. Ch, chicken strain of *C. jejuni*; Th, song thrush strain of *C. jejuni*; C, control; AE, algal extract. Red = Ch + AE, Green = Ch + C, Blue = Th + AE, Purple = Th + C.

All birds were *Campylobacter jejuni* negative prior to the challenge and successful *C. jejuni* colonization in both strains was achieved after the challenge at 1 dpi (18 days of age). There was no significant effect of feed supplementation with *AE* on the colonization dynamics of either of the *C. jejuni* strains, as determined by CFU counts on agar plates (Figure 1). The levels of *C. jejuni* in birds that received *AE* in their diet (Ch + *AE* and Th + *AE*) were similar to *C. jejuni* levels in groups fed with the *C* diet (Ch + *C* and Th + *C*) at all sampling points. No significant differences were observed between the chicken and song thrush *C. jejuni* strains in their ability to colonize the broilers' gut, and similar colonization levels were observed from dpi 1 to dpi 19 in both *C* and *AE*-fed birds. *C. jejuni* colonization progressed rapidly from dpi 1 to dpi 2, and the peak was observed 3 days after the challenge (3 dpi) with ~10^7^ CFU/g of feces. Thereafter, the colonization intensity for both strains remained at similar levels of 10^6^ CFU/g of feces on average until the end of the experiment.

### 3.2 Caecal microbiota composition shifts

Rarefaction curves of observed amplicon sequencing variants (ASVs) in caecal samples revealed sufficient sequencing depth to capture species richness at days 7, 14, and 37 after hatching (Supplementary Figure 1). The alpha diversity, as measured by the observed number of ASVs had the lowest count at day 7, followed by day 14, and the highest number of ASVs was present at day 37.

**Figure 2:**
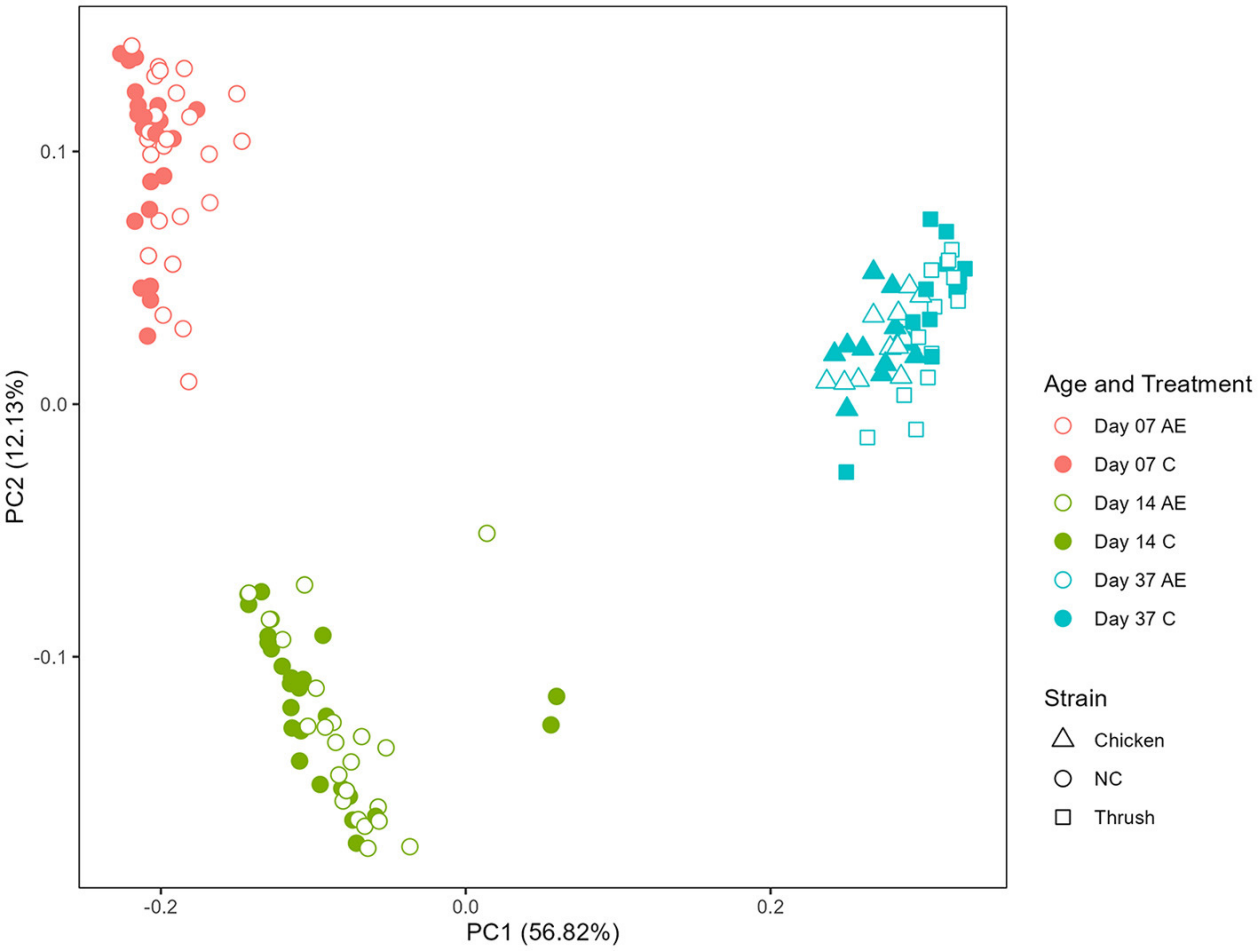
Principal coordinate analysis (PCoA) plot showing differences in generalized UniFrac distance matrix at different sampling points after hatching (day 7, day 14, and day 37). AE, algal extract; C, control; NC, non-challenged.

Principal coordinate analysis (PCoA) was used to display differences in the generalized UniFrac distance matrix at different sampling points after hatching (day 7, day 14, and day 37). The results demonstrate a distinct ASV clustering according to time after hatching (Kruskal-Wallis, *p* < 0.001) (Figure 2). The differences between sampling points day 7 and day 14 indicate an effect of age, whereas the microbiota composition at day 37 could be affected by age, cage effect or *C. jejuni* colonization (PERMANOVA, *p* = 0.001). No clear separation between groups fed with *AE* supplemented diet in comparison to the *C* groups was observed, indicating that the caecal microbiota composition was not significantly affected by supplementation with *AE*.

**Figure 3:**
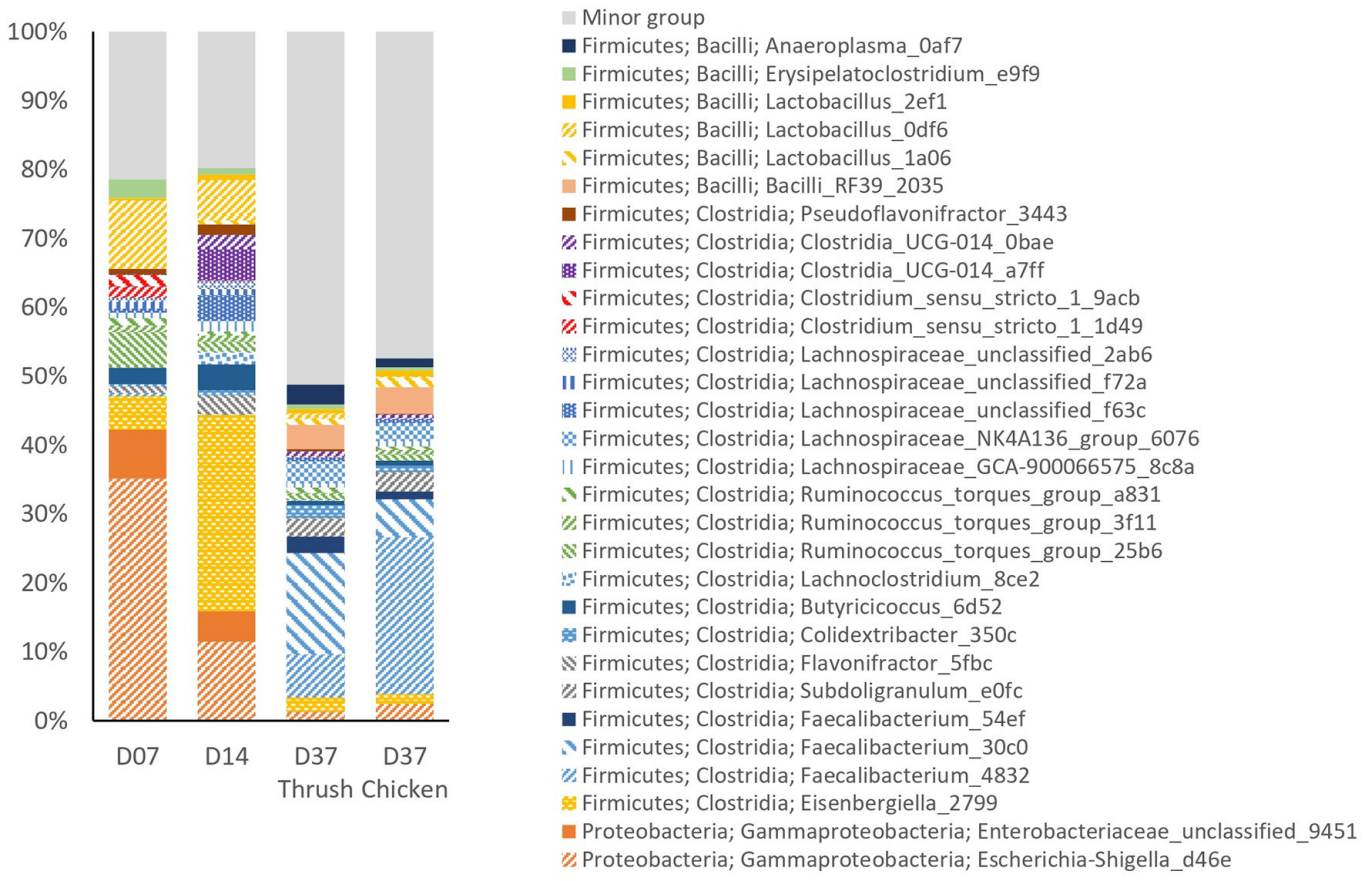
The relative abundance (%) of the top 30 most abundant ASVs in caecal samples at different ages (day 7, day 14, and day 37) and *Campylobacter* strains (song thrush and chicken).

The relative abundance (RA) of the top 30 most prevalent ASVs in the caeca (Figure 3) accounted for ~80% of the total sequencing reads at day 7 and day 14. At day 37, this figure had dropped down to ~50%, concurrent with a substantial increase in the number of low abundant ASVs in the minor group; from 89 on day 14 to 285 and 297 on day 37 for the group infected with the thrush strain or the chicken strain, respectively. This minor group describes the group of ASVs, with each having an abundance lower than 0.55%. Since the caecal microbiota development was not significantly affected by supplementation with *AE*, the treatments were pooled for further analysis. In general, there was considerable individual variation in the caecal microbiota among the birds observed.

Examination of taxonomic composition revealed shifts in the caecal microbiota composition between the sampling points. Comparison of pre-*C. jejuni* challenge samples from day 7 and day 14 showed a considerable decrease in the RA of ASV *Escherichia-Shigella*_d46e at day 14, while *Eisenbergiella*_2799 showed a pronounced increase. At day 37 (post-challenge samples), the RA of both *Escherichia-Shigella*_d46e and *Eisenbergiella*_2799 had decreased to low percentages. In contrast, *Faecalibacterium*_4832 and *Faecalibacterium*_30c0 emerged as the dominant ASVs at day 37, with a minor representation at days 7 and 14. Unclassified *Enterobacteriaceae*_9451 showed the highest RA at day 7, with a decrease at day 14, and minimal presence at day 37. ASVs *Clostridium*_*sensu*_*stricto*_1_1d49 and *Clostridium*_*sensu*_*stricto*_1_9acb were present at day 7 and thereafter their RA was minimal. Conversely, ASVs *Clostridia*_UCG-014_a7ff and *Clostridia*_UCG-014_0bae were the most abundant at day 14 and diminished at day 37. The most abundant ASV classified as *Lactobacillus* was *Lactobacillus*_0df6, which had the highest RA at day 7, decreased by day 14, and was minimally present at day 37. Between day 14 and day 37, there was a distinct increase in the abundance of *Faecalibacterium*_54ef, *Bacilli*_RF39_2035, and *Anaeroplasma*_0af7. These ASVs were not present among the top 30 most abundant ASVs at day 7. The ASV *Subdoligranulum*_e0fc exhibited minor presence at days 7 and 14, with a considerable increase in its RA at day 37. A reduction in the RA of *Butyricicoccus*_6d52 was noted at day 37 in both groups (chicken and thrush strain), in contrast to its moderate abundance at days 7 and 14.

**Figure 4:**
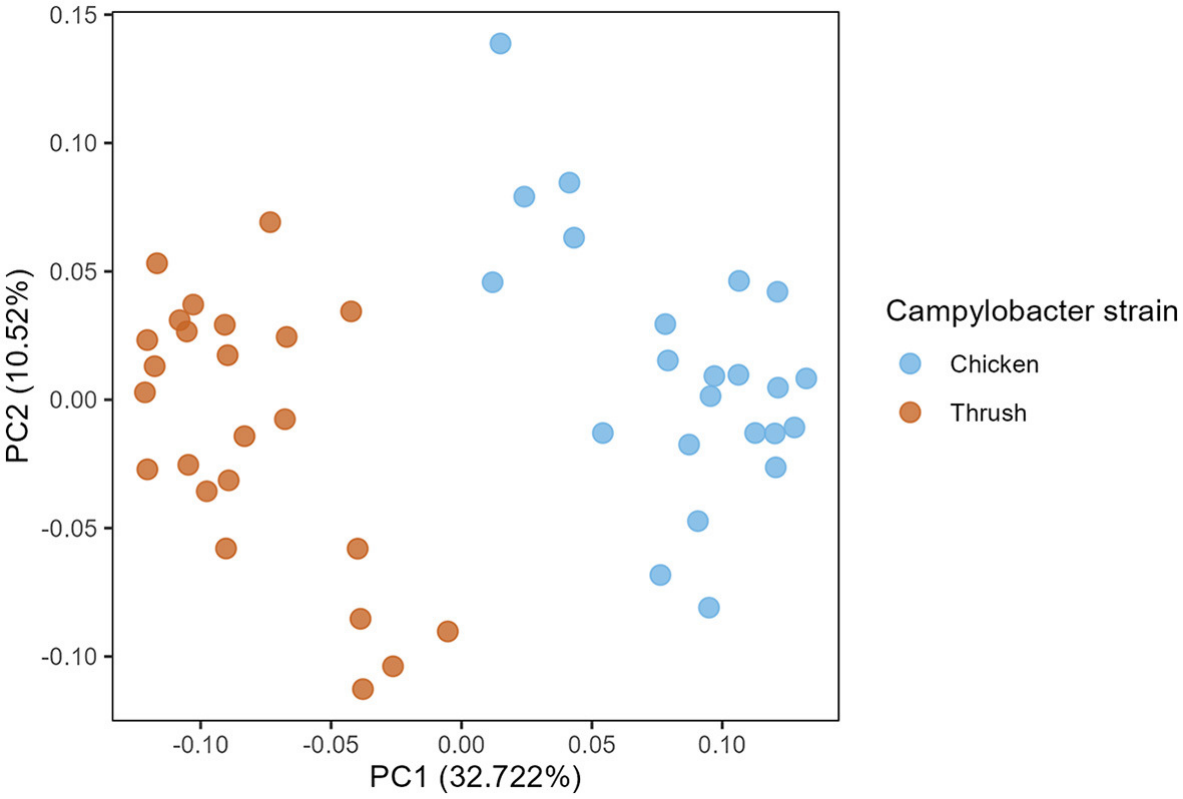
Principal coordinate analysis (PCoA) plot showing differences in generalized UniFrac distance matrix between different *Campylobacter* strains (chicken and song thrush origin) at age day 37.

A PCoA plot of caecal samples obtained at day 37 after hatching revealed a significant clustering according to the *C. jejuni* strain used for the challenge (PERMANOVA, *p* = 0.001) (Figure 4). No effect of diet was observed at day 37.

**Figure 5:**
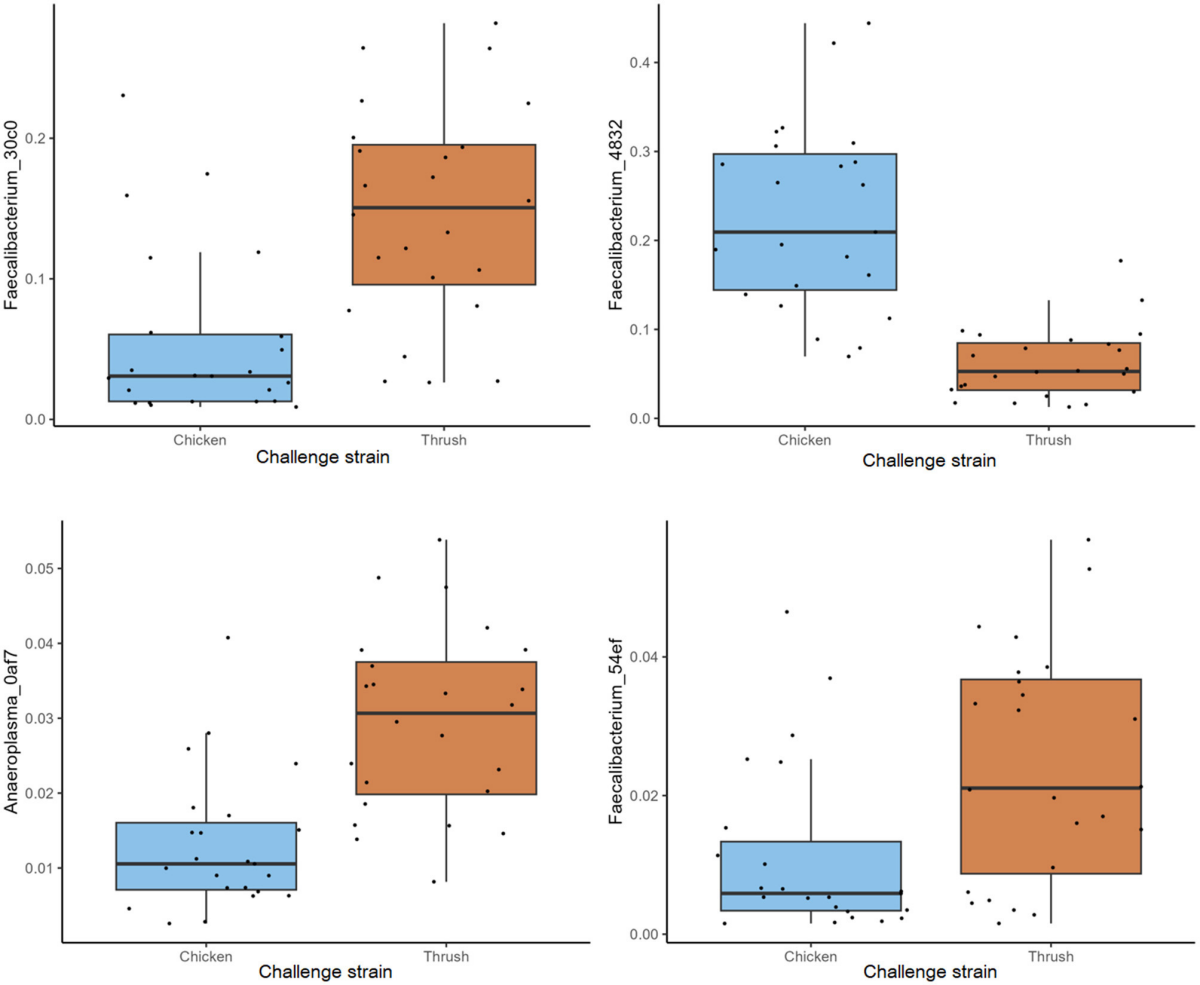
The relative abundance (%) of ASVs in caecal samples at day 37 with a significant difference (*p* < 0.05).

At day 37, a significant difference (*p* < 0.05) in the RA of several ASVs was detected in caecal samples obtained from broilers challenged with the two different *C. jejuni* strains. The most abundant ASV within the group challenged with the song thrush strain was *Faecalibacterium*_30c0, exhibiting a significantly higher RA compared to the chicken strain samples (Figure 5). In contrast, the dominant ASV found in the samples from birds challenged with the chicken strain was *Faecalibacterium_*4832. Furthermore, a significantly higher abundance of ASVs *Anaeroplasma*_0af7 and *Faecalibacterium*_54ef was observed in the thrush strain samples than in the chicken strain samples (Figure 5). We cannot exclude that these differences were due to environmental factors as the birds challenged with the two different strains were caged in different parts of the stable, separated by a flexible wall.

## 4 Discussion

Identifying ways to reduce *Campylobacter jejuni* colonization in broilers' gut is desirable to minimize the risk of meat contamination during the slaughter process and thereby enhance the safety of poultry products for human consumption. Feed supplementation with prebiotics and probiotics may be an important component of the complex solution to this challenge.

Our study's results revealed that supplementation with 290 ppm laminarin had no significant effect on the colonization dynamics of two distinct strains of *C. jejuni*: the chicken origin strain #65 and the song thrush origin strain #3926. No significant differences in bacterial loads could be observed between the groups that received laminarin supplementation compared to the control groups at any time point during the study (Figure 1). These findings are in line with an earlier study by Sweeney and O'Doherty (2016), where the addition of purified laminarin extract (250 ppm) to the diet of broilers showed no significant impact on the suppression of *C. jejuni* during the initial post-hatch period. Taken together, this suggests that laminarin-rich algal extracts have a limited direct antimicrobial effect on reducing *C. jejuni* colonization in broiler caeca.

The chicken and song thrush origin *C. jejuni* strains exhibited comparable colonization dynamics during the whole experiment (Figure 1), indicating that the Ross 308 hybrid used in this study displayed similar susceptibility to colonization by both *C. jejuni* strains. This observation was in contrast to the study by Atterby et al. (2018) where dramatic differences in colonization patterns were observed in mallard ducks inoculated with either the chicken strain #65 or the song thrush strain #3926, both used in this study. In wild birds, *C. jejuni* shows strong host association that might be due to colonization resistance to specific *C. jejuni* genotypes (Griekspoor et al., 2013; Atterby et al., 2018). Our findings suggest that such colonization resistance against specific *C. jejuni* genotypes might be weaker in broiler chickens. However, further investigation utilizing a broader range of *C. jejuni* strains from diverse bird host origins is necessary to verify this. The fast establishment and continuous persistence of colonization observed in both strains highlight the efficiency with which *C. jejuni* establishes itself in the broilers' caeca. Colonization levels remained high from 3 dpi until the end of the experiment. These observations are in agreement with the results reported by Connerton et al. (2018), who documented the onset of *C. jejuni* colonization in broilers infected at 20 days of age within 2 days of exposure, with colonization persistence until the end of the study at day 35.

The caeca are known to be the most bacteria-dense part of the broiler's gastrointestinal tract and an important site for carbohydrate fermentation contributing to the birds' intestinal health and nutrition (Waite and Taylor, 2014). To evaluate the impact of diet supplementation with *AE* on broiler microbiota development, caecal samples were analyzed by 16S rRNA amplicon sequencing and investigated at the ASV level. Principal Coordinate Analysis (Figure 2) used to visualize the dissimilarities within the generalized UniFrac distance matrix across different sampling points (day 7, day 14, and day 37) did not show any clear clustering of the groups that received feed supplemented with *AE* compared to the control groups. This suggests that the supplementation did not significantly influence the composition of the caecal microbiota in the current experiment. Yet, Venardou et al. (2021) found that supplementing the basal diet with 300 ppm of laminarin for 35 days led to an increase in both the relative and absolute abundance of *Bifidobacterium* in the caeca of Ross 308 broiler chickens, proposing this dosage to promote a beneficial gastrointestinal microbiota profile. However, it is worth mentioning that in that study broilers were not exposed to experimental *Campylobacter* challenge and that the influence of dietary supplements can vary based on diverse factors such as host physiological state, application pattern, housing effect, and dosage, which can yield different outcomes in their activity (Marco and Tachon, 2013; Salami et al., 2016). Additionally, pronounced individual variation observed in the caecal microbiota composition among the birds on all sampling points in the present study highlights the complexity of avian gut microbial communities (Stanley et al., 2013).

The age of the birds exerted a significant effect on the microbiota composition, as suggested by the distinct clustering of ASVs from each sampling point, correlating to the age of the birds (Figure 2). Furthermore, the rarefaction curves of observed ASVs revealed a consistent temporal increase in alpha diversity (Supplementary Figure 1). These observations find support in previous studies which have highlighted the age-dependent aspect of caecal microbiota development (Ivarsson et al., 2022; Boyner et al., 2023). Among changes in the gut microbiota solely connected to the age of the birds, a relatively high RA of ASVs *Clostridium_sensu_stricto*_1 as well as ASVs *Escherichia-Shigella*_d46e and unclassified *Enterobacteriaceae*_9451 (belonging to the phylum Proteobacteria), were observed at day 7, while their RA notably decreased at day 14 (Figure 3). Our observation aligns with a study conducted by Kers et al. (2020), suggesting that members of the genus *Clostridium sensu stricto* are among the first colonizers of the avian caeca, and that after a few days, members of the phylum Proteobacteria colonize the caeca, followed by an increase in Firmicutes later in the broiler's life.

Since the main aim of this study was to investigate if feeding broilers with *AE* had an effect on their resistance to *C. jejuni* colonization and if this could be explained by their caecal microbiota composition in contrast to birds fed control feed, we did not include a control group that was not challenged with *C. jejuni*. Regrettably, this fact renders us unable to draw direct conclusions on the relative effect of *C. jejuni* colonization vs. that of bird age on the caecal microbiota composition observed at day 37. However, this study provides valuable insights into caecal microbiota development during *C. jejuni* challenge, focusing on the ASV level rather than the higher taxonomic levels commonly discussed in broiler gut microbiota studies. Interestingly, the challenge with the two different *C. jejuni* strains at day 17 affected caecal microbiota composition differently by day 37 (Figure 5), suggesting an influence of *C. jejuni* colonization. ASVs classified as *Faecalibacterium* were pronounced at day 37, where ASV *Faecalibacterium*_4832 dominated in birds challenged with the chicken strain, while ASVs *Faecalibacterium*_30c0 and *Faecalibacterium*_54ef had significantly higher RA in the birds that received the song thrush strain. These findings are consistent with prior studies by Ocejo et al. (2019) and Thibodeau et al. (2015), who reported a positive association between *Faecalibacterium* and *Campylobacter* presence in broiler caeca. Moreover, similarities to our previous research (Valečková et al., 2023) were found, where *Faecalibacterium* emerged as the predominant genus in Ross 308 caeca 20 days after *C. jejuni* challenge. Furthermore, Liao et al. (2020) noted that *Faecalibacterium* dominated the caecal bacterial communities of *Campylobacter*-free broilers during the grower phase (7-14 days of age), while its prevalence decreased thereafter. In addition, in the current study, significantly higher RA of *Anaeroplasma*_0af7 was observed in birds that received the song thrush strain. Current reports about *Anaeroplasma* in broilers caeca in relation to *Campylobacter* presence are limited. However, Ocejo et al. (2019) identified *Anaeroplasma* as the main genus of the minor phyla Tenericutes, which was present at stable levels (0.002%−1.5%) throughout the productive period when analyzing the microbiome of chicken caeca in two different breeds (Ross-308 and Sasso-T451A), and management systems throughout their whole productive lifespan. It should be noted that the differences at ASV level seen at day 37 may also be due to environmental effects imposed by the fact that birds challenged with the two different *C. jejuni* strains were physically separated in the stable by a flexible wall (Kers et al., 2018).

Analyzing the RA of the top 30 most abundant ASVs revealed the pronounced reduction of ASV *Escherichia-Shigella*_d46e after the *C. jejuni* challenge. This observation aligns with results from Awad et al. (2016), where 14-day-old Ross 308 broilers were challenged with 1 × 10^8^ CFU of *C. jejuni* NCTC 12744 and their microbial community composition was followed from day 1 to day 28 of age. The authors found that the presence of *C. jejuni* was associated with a significant reduction in *Escherichia coli* abundance compared to the non-challenged control birds. These findings may also be related to the observed decrease in RA of unclassified *Enterobacteriaceae*_9451 at day 37 in the present study. Furthermore, the RA of ASVs *Clostridia* UCG-014 decreased considerably post-challenge which aligns with the results from Pang et al. (2023), where a significant negative correlation between *Campylobacter* abundance and genera *Clostridia* was reported in caeca of 5 weeks old broilers. Additionally, a considerable increase in the RA of ASVs *Subdoligranulum*_e0fc and *Bacilli*_RF39_2035 at day 37, compared to their minimal presence prior to the challenge, along with a converse shift in the RA of *Eisenbergiella*_2799, *Butyricicoccus*_6d52, and *Lactobacillus*_0df6 in response to the challenge, aligns with our previous findings discussing the effect of *C. jejuni* presence on the broilers caeca microbial composition in Ross 308 (Valečková et al., 2023).

The observed increase in the species richness within the minor ASV group (Supplementary Figure 1) and the increased RA of ASVs within this group at day 37 (Figure 3) was likely attributed to the progressive divergence in the caecal microbiota with age (Zhou et al., 2021). Ocejo et al. (2019) identified age as the strongest factor influencing the composition of caecal microbiota when the core microbiome was compared among Ross 308 broilers raised in a conventional system for 42 days with that of a 'slow-growing breed', Sasso T451A reared in an extensive farming system with outdoor access for 86-days. Interestingly, the same study reported different microbial taxa to be either positively or negatively correlated with *C. jejuni* RA, suggesting that both bird age and *C. jejuni* colonization can affect the caecal microbiota composition. Therefore, it can be speculated that the drastic increase in species richness observed on day 37 could have been an effect of the experimental *C. jejuni* challenge, where the host immune response triggered by *C. jejuni* colonization might selectively have promoted the profusion and growth of specific ASVs, resulting in an increase in microbial diversity as suggested in a study conducted by Pang et al. (2023). In their analysis of samples collected from five-week-old birds, the researchers observed that farms with *Campylobacter*-positive broilers had a higher species richness in the caecal microbiota and greater phylogenetic diversity in comparison to *Campylobacter*-negative farms. As stated above, it is important to note that the interpretation of pre- and post-challenge observations in the current study is obscured by the considerable age difference between the two sampling occasions, specifically 23 days. Further studies are therefore required to better understand changes in caecal microbiota composition related to *C. jejuni* colonization in general and the presence of different *C. jejuni* strains in particular.

In conclusion, the present study demonstrates that the supplementation of *AE* in broiler diets has no significant impact on either *C. jejuni* colonization after experimental challenge or the development of the caecal microbiota. The results suggest that the overall changes in the gut microbiota observed at different sampling points were mainly age-dependent. Furthermore, the results do not support the initial hypothesis that the chicken origin *C. jejuni* strain would exhibit better colonization ability in chickens compared to the strain originating from a song thrush. Yet, this study contributes to understanding the complex interactions influencing microbial communities in the broiler gut and provides insights for future interventions aimed at enhancing resistance to *Campylobacter* colonization in broilers.

## Data availability statement

The sequencing data are deposited in NCBI, accession number PRJNA1078836.

## Ethics statement

The animal study was approved by the Uppsala Ethics Committee for Animal Research, Uppsala, Sweden approval number (5.8.18-10572/2019). The study was conducted in accordance with the local legislation and institutional requirements.

## Author contributions

EE: Writing – review & editing, Writing – original draft, Visualization, Investigation. LS: Writing – review & editing, Writing – original draft, Visualization, Validation, Software, Methodology, Formal analysis, Data curation. GC: Writing – review & editing, Validation, Resources. HP: Writing – review & editing, Resources, Funding acquisition. GT: Writing – review & editing, Validation, Methodology, Investigation, Data curation. PE: Writing – review & editing, Writing – original draft, Validation, Supervision, Resources, Methodology, Investigation, Funding acquisition, Conceptualization. EI: Writing – review & editing, Supervision, Project administration, Investigation, Funding acquisition, Conceptualization.
