# Supplementary material for: No colonization resistance to Campylobacter jejuni in broilers fed brown algal extract-supplemented diets
Source: Front Microbiol. 2024 Jun 27;15:1396949. doi: 10.3389/fmicb.2024.1396949 (PMC11236747; doi:10.3389/fmicb.2024.1396949)
Supplement: Supplementary file 1 [file Table_1.DOCX]

Supplementary Table 1. Effect of treatments on feed intake (FI; g/chicken), accumulated BW (g/chicken), and calculated feed conversion ratio (FCR), based on weekly group weight of birds and feed. Least square means and pooled standard error of means (SEM).

|  | Treatment^1^ | |  |  |
| --- | --- | --- | --- | --- |
|  | C | AE | SEM | P-Value |
| **BW** |  |  |  |  |
| d6 | 142.5 | 151.7 | 3.27 | 0.0600 |
| d13 | 454.8 | 471.8 | 9.19 | 0.2041 |
| d20 | 1015.8 | 1050.5 | 30.01 | 0.2570 |
| d27 | 1639.0 | 1727.3 | 46.30 | 0.0805 |
| d34 | 2454.4 | 2579.8 | 56.54 | 0.0716 |
| d37 | 2975.5 | 3090.7 | 60.64 | 0.1327 |
|  |  |  |  |  |
| **FI** |  |  |  |  |
| d6 | 96.4 | 105.2 | 3.23 | 0.0664 |
| d13 | 355.5 | 376.1 | 10.88 | 0.1952 |
| d20 | 1361.7 | 1397.1 | 22.51 | 0.2785 |
| d27 | 2293.2 | 2381.3 | 40.59 | 0.1401 |
| d34 | 3542.3 | 3667.8 | 57.90 | 0.1410 |
| d37 | 4422.6 | 4533.3 | 69.58 | 0.2741 |
|  |  |  |  |  |
| **FCR** |  |  |  |  |
| d6 | 1.01 | 1.01 | 0.015 | 0.8424 |
| d13 | 0.85 | 0.86 | 0.018 | 0.4229 |
| d20 | 1.40 | 1.39 | 0.042 | 0.8225 |
| d27 | 1.41 | 1.38 | 0.023 | 0.1957 |
| d34 | 1.43 | 1.41 | 0.017 | 0.0839 |
| d37 | 1.48 | 1.45 | 0.016 | 0.0892 |

Abbreviations: C = control diet; AE = a diet supplemented with algal extract.

^1^Treatment = chicken diet.
