# Supplementary figures and images for: No colonization resistance to Campylobacter jejuni in broilers fed brown algal extract-supplemented diets
Source: Front Microbiol. 2024 Jun 27;15:1396949. doi: 10.3389/fmicb.2024.1396949 (PMC11236747; doi:10.3389/fmicb.2024.1396949)

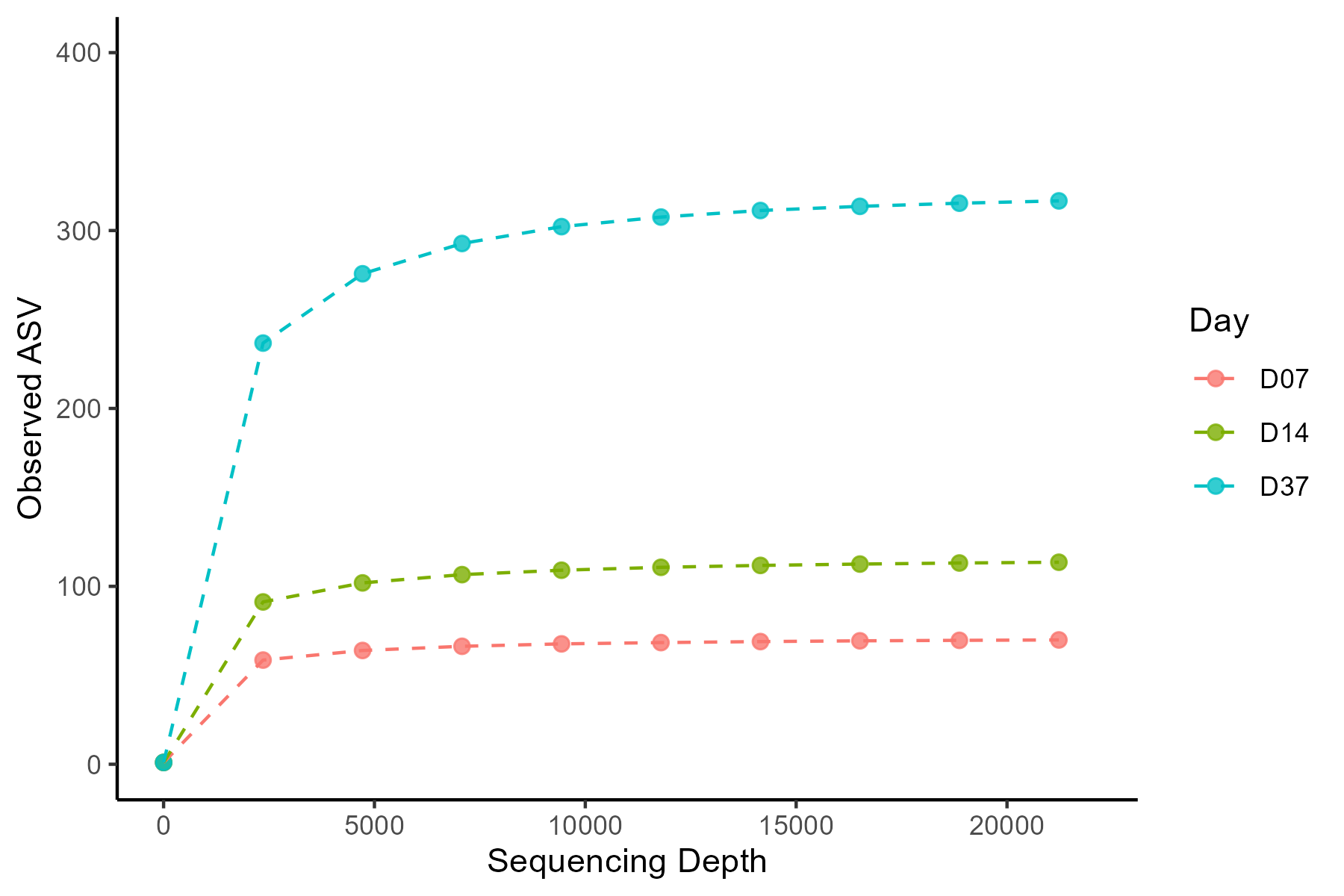

Supplement: Supplementary file 2 [file Image_1.TIFF]
